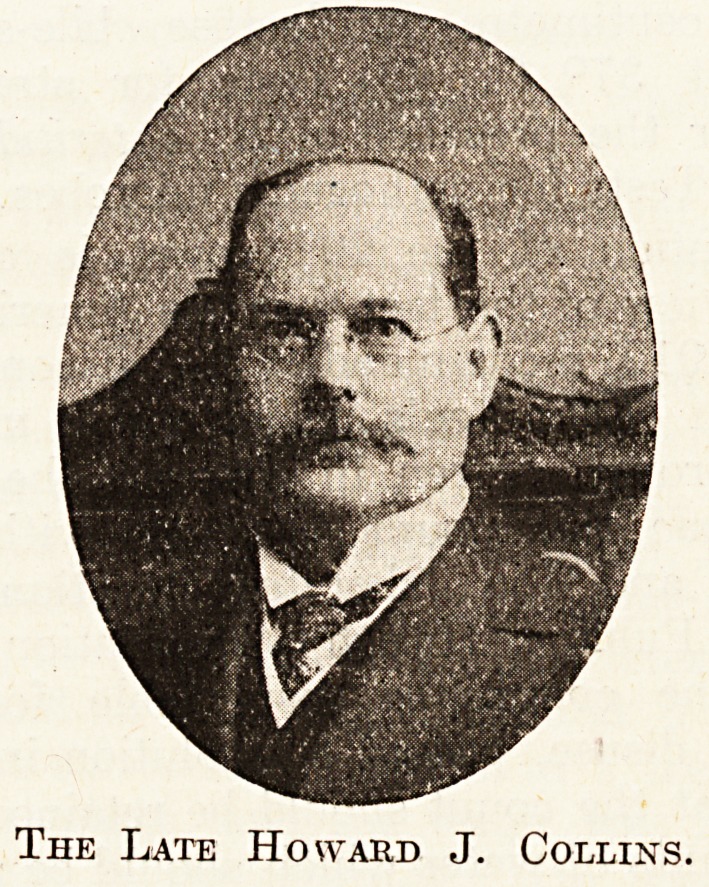# The Death of Mr. Howard J. Collins

**Published:** 1914-10-24

**Authors:** 


### THE DEATH OF MR. HOWARD J. COLLINS.

### A Memoir by One Who Knew Him Well.

The death of Mr. Howard J. Collins, house governor and secretary of the Birmingham General Hospital, whose illness (cancer of the rectum) was recorded in The Hospital of September 26, took place on Saturday last, and removes one of the best known men in the hospital world.

Mr. Collins, who was born at Poplar in 1858, commenced his career in the accountant's department of the East Indian Railway Company, from where he was made accountant to the Southern Mahratta Railway Company, London, after which he was appointed successively assistant secretary and accountant of the London Lock Hospital and Asylum, secretary of the Hospitals Association, London, secretary and house steward of Norfolk and Norwich Hospital, Norwich, and house governor and secretary of the Birmingham General Hospital.

His Work in Birmingham.

Mr. Collins, appointed in March 1892, went to the Birmingham General Hospital at a busy time. The site for the new general hospital in Steelhouse Lane (the foundation-stone of which was laid on September 8, 1894, by their Majesties, the King and Queen when Duke and Duchess of York) was taken in hand the year following his appointment. An Act of Parliament was also obtained extending the period for claiming Miss Rylands' bequest of £25,000. He superintended the removal from the old building, which commenced on October 16, 1897, when the eighty-six patients were transferred from Summer Lane to the new hospital.

His administration at this time was conspicuously successful, and won the admiration of the board and medical staff. Mr. Collins was a Fellow of the Royal Statistical Society, a Fellow of the Chartered Institute of Secretaries, a member of the Council of the British Hospitals Association—for which he was local conference secretary in 1912, when the meetings of the association were held in Birmingham; president of the Birmingham and Midland Society of East Anglians; at one time secretary of the Birmingham Triennial Musical Festival, and president of the Birmingham Photographic Society. He founded the Charity Secretaries' Club—consisting of secretaries or other principal administrative officers of hospitals and charities within twenty-five miles of Birmingham. He was also a prominent Freemason.

The Late Howard J. Collins.

On July 4, 1908, at St. Peter's Church, Lee, Kent, Mr. Collins married Miss Hilda Sandham Dunstan, daughter of the late Arthur Proctor Dunstan and Mrs. Dunstan, of "Fairholm," Eltham Road, Lee, who survives him, and for whom the sincerest sympathy will be felt in her bereavement. Another aspect of Mr. Collins' work and personality will be found touched on in our Notes this week.

## Figures and Tables

**Figure f1:**